# Nurturing connections: Patient insights on online resources for cancer information and support

**DOI:** 10.1177/20552076261475254

**Published:** 2026-09-17

**Authors:** Terese Knoppers, Sarah Bouhouita-Guermech, Ana Eliza Bonilha, Angelica Voutsinas, Marie Cosquer, Ma’n H. Zawati

**Affiliations:** 1 Centre of Genomics and Policy, 574124 McGill University, Montreal, QC, Canada

**Keywords:** cancer, online resources, web portals, information, support, holistic, patients, qualitative

## Abstract

**Objective:**

The present study aimed to better understand the views of people living with or beyond cancer in Canada on the role and potential of online resources for cancer-related information and support (CRIS).

**Methods:**

This study was guided by a descriptive exploratory qualitative design and employed a patient-centered lens. In total, 50 adults from across Canada took part in online focus groups of two to five participants each. Four members of our research team collaborated on content analysis of the focus group transcripts; iteratively engaging themes derived from the data. Our Analysis and Discussion centrally attend to how agency is exercised, enabled, and/or elided for participants in their use of the internet for CRIS.

**Results:**

Participant engagement produced three interconnected themes: (1) seeking support: reasons for and factors in internet use for CRIS, (2) navigating the digital landscape: gaps, barriers and strategic approaches, and (3) future horizons: ideas and ideals for a Pan-Canadian therapeutic web portal for patients.

**Conclusions:**

Findings contribute patient perspectives to two bodies of literature: on patient use of the internet for CRIS and on the development and potential of therapeutic web portals. Results can help web developers, clinicians, researchers, and policymakers be sensitive and responsive to the informational and support needs of people living with or beyond cancer.

## Introduction

Cancer diagnosis is a complex, life-transformative experience with physical, psychosocial, and practical reverberations.^1,2^ It is also increasingly common, as two in five people can expect a cancer diagnosis in their lifetime,^3^ and increasingly something people are living with or beyond, as detection, prognosis, and treatment improve for many cancers.^4–6^ Just as the experience of cancer is multidimensional, it brings multidimensional questions and needs regarding care and wellbeing.^1,2^ People reckon with the reality of cancer within their lives, navigate multiple medical providers, interventions, and facilities, experience changes in their wellbeing and capacity, grapple with unknowns, and seek answers and support. While healthcare providers (HCPs) remain the preferred source of information for people diagnosed with cancer, for a variety of reasons patient needs are not necessarily met by HCPs alone.^7–12^ More and more people are turning to the internet as a source of cancer-related information and support (CRIS).

A substantive body of literature has chronicled the importance and increasing centrality of the internet in the lives of people diagnosed with cancer over the past three decades. Indeed, the percentage of cancer patients using the internet for CRIS rose from a small minority in the 1990s to a strong majority in the 2010s.^13^ This shift was driven by growing household internet access, increased variety and volume of online resources, the internet’s relative accessibility and convenience, and the trend towards patients seeking greater agency in their own healthcare odysseys.^13,14^ More recently, there has been another shift wherein older adults are engaging with the internet for cancer information and support at significantly higher rates. In Canada, 83% of those aged 65 and older were internet users as of 2020, up from 46% in 2010.^15^ Adults 65 and older are the fastest growing population of internet users in the country, and people aged 50-64 are the second fastest. As nine out of ten people diagnosed with cancer are aged 50 or older,^15^ the influx of older users brings new considerations to online cancer resource design and delivery and indicates a transformed landscape for CRIS in which the internet has become a significant feature for people of all ages.^16^

There is a considerable range in the form, quality, and nature of online cancer resources. Alongside use of the open web for CRIS, there has been increasing attention to the value of structured online tools and venues for clinical information and support.^17,18^ Structured online resources include cancer and patient organizations, government agencies, healthcare and academic institutions, commercial businesses, and peer support forums, among others. One specialized resource has been the emergence of patient and therapeutic web portals. Patient portals house individual health information and facilitate clinical interaction and follow-up, enabling more active patient participation, more efficient service access, and better continuity of care.^19–23^ Therapeutic portals consolidate and share clinical research health data, findings, and information related to a specific health condition, increasing the speed and reach of new knowledge and facilitating its flow from bench to bedside and back again.^23–25^ Both forms of web portals hold important potential to improve health outcomes and facilitate patient empowerment. Of the two types of portals, patient portals are more common and relatively established,^26^ whereas therapeutic portals are newer, and their optimal design, use, and utility are just beginning to be explored.^23–25^

Existing literature on internet engagement by people living with and beyond cancer is centrally concerned with reasons for use, user demographics, health literacy, usage patterns, quality of information, and the impacts of engagement on the relationship with healthcare providers and decision-making.^13,14^ Research has shown that people diagnosed with cancer use the internet for a variety of reasons, including to better understand and help cope with their diagnosis, treatments, and side-effects, to get help with decision-making and prepare for consultations with HCPs, to reinstill a sense of agency and reduce uncertainty through knowledge, to reduce isolation, and to find peer support.^7,27–31^ High quality online information has been found to improve mental wellbeing, quality of life, and to ameliorate the patient experience by shoring up patient skills, agency, and expertise.^6,12,29,30,32,33^

At the same time, age, education, income, gender, proximity to urban centers, cancer type and stage, and degree of family/social support all impact the nature of internet use on the part of cancer patients, suggesting that the internet is more helpful for some than others.^2,34–36^ These differences reflect a broader digital divide in Canada, where socioeconomic disadvantage, geographic isolation and limited digital literacy restrict access to online health information and support, resulting in inequitable opportunities to benefit from digital cancer resources.^35,37–39^ There are also considerable concerns about the preponderance of online information that is incorrect or potentially harmful.^37,40–43^ In terms of the HCP-patient relationship, the literature discusses how structured online resources and venues have played a key role in the transition from more asymmetric paternalistic models of health communication towards person-centered shared decision-making models.^13,44^ However, patients and HCPs alike have been found to be reluctant to discuss general internet use for CRIS with each other, and this lack of active communication has been characterized as a key obstacle in e-health.^13,34^

While descriptive aspects of cancer-related internet use and the perspectives of researchers and clinicians have been well characterized, there is a relative lack of patient-centered literature on the subject.^14,42^ Less attention has been paid to what people diagnosed with cancer want, need, and hope for from the internet, how it does and does not fulfill the above, and the concrete ways it could. Patient perspectives are integral to the design and implementation of relevant, accessible, and effective cancer web resources. The present study aimed to better understand the views of people living with or beyond cancer on the role of online resources for cancer information and support. Qualitative focus groups covered a broad range of material distilled into three topics: use of and relationship to the internet for cancer-related information and support, gaps and barriers participants encountered, and ideals for content and features on a Canadian therapeutic web portal dedicated to CRIS. This research contributes to two streams of literature: on the use of the internet for CRIS and on the role and potential of therapeutic web portals for patients. It fills a gap where there is a need for further understanding of patient perspectives on both.^10,14,17,24,25,42^ Specifically, it advances existing knowledge by centering patient perspectives and agency in both current online resource use and the envisioned design, content, and functions of therapeutic portals - moving beyond descriptive accounts toward actionable insights for patient-centered digital resource development. Findings can be applied to help web developers, clinicians, researchers, and policymakers be sensitive and responsive to the informational and support needs of people living with or beyond cancer.

## Methods

### Study design

This study employed an exploratory contextualist qualitative design.^45^ It consisted of semi-structured focus groups analyzed using descriptive content analysis and guided by a conceptual lens that centers patient agency. Qualitative methods were appropriate to enable an in-depth, nuanced exploration of participants’ perspectives and experiences in an area where stakeholder views remain underexplored.^46^ Semi-structured focus groups were selected to both address the research questions and allow participant-driven concerns and priorities to emerge.^47,48^ Descriptive content analysis was appropriate to summarize the perspectives of participants across the data set and because of the practical focus of the research aims.^49,50^ Ethics approval for the study was granted by the Research Ethics Board of McGill University’s Faculty of Medicine and Health Sciences (IRB# A12-B94-21B (21-12-016)). Methods and findings have been reported in light of the Standards for Reporting Qualitative Research (SRQR) checklist.^51^

### Reflexivity statement

This qualitative engagement research study is one component of a larger research project exploring the potential of therapeutic portals led by the senior author. The broader research program also included engagement with researchers and clinicians regarding the design and implementation of such platforms.^25^ The research team is based out of the Centre of Genomics and Policy at McGill University in Montreal, Quebec, with authors’ disciplines spanning counseling psychology, sociology, bioethics, human genetics, law, and political science. The authors also have varied lived experiences related to cancer and online resources as a potential source of information and support which they bring to their work in this area. These experiential knowledges and disciplinary perspectives, as well as the Centre’s longstanding interest in responsible innovation and implementation of emerging health technologies, inevitably shaped the questions we asked and the issues to which we were attentive during research design, data collection, and analysis.

### Conceptual framework

Our conceptual framework guided the study by serving as a sensitizing lens to the issue of patient agency during all stages of the research process.^52^ Our framework adapts Shim’s (2022) theory of patient agency.^53^ Citing research that shows the health benefits of a sense of control, of empowerment to reflect one’s views on medical treatments, and of integrating and processing illness into one’s lifeworld and subjectivity, Shim argues that regardless of eventual health outcomes, personal agency is essential to people in illness. Shim writes: “the regain of one’s biography and a sense of living, either through the recovery of health or the adaptions to illness, becomes the paramount issue” (p. 2). Following a review of qualitative studies of experiences of illness, Shim proposes holistically attending to practices of agency people with illness use to continue to lead a meaningful life despite significant discontinuities and disruptions. This can include reworking physical/bodily control, shifting focus between or embedding agency in multiple dimensions of life (e.g. physical, psychological, social and/or religious/spiritual) or, when health problems preclude control over life circumstances, by adapting to these circumstances and exercising dignity and agency in end of life.

Given the study aims and the specificity of cancer as an illness, our conceptual framework centrally attends to how agency is exercised, experienced, enabled, and/or elided for participants in their relationship to the internet as a source for cancer information and support. Rather than reducing people to their medical condition or projecting a singular experience of cancer, the focus is on people as active agents with multi-faceted lives and for whom, once cancer enters their lives, enact agency in multi-faceted ways. We are interested in what the internet does for people in their relationship to cancer in their lives, as well as what it could do ideally.

### Recruitment and study participants

The study methods leads have extensive prior qualitative experience, and collaborated on the question template and ethics application. Recruitment occurred via convenience sampling. The criteria for study inclusion were that potential participants be legal adults who had received cancer diagnoses and were presently in treatment or recovery in Canada. The recruitment ad was circulated via Canadian cancer organization and association websites, social network pages (Facebook and Instagram), and member email lists. Interested participants contacted the team via email, and completed an initial assessment form to ensure they met the inclusion criteria, as well as a sociodemographic survey. Interested participants who met the study criteria were then emailed more information about the study, the consent form to read and sign, and invited to ask any questions they may have over email or phone. Written consent was established prior to study enrollment. Focus groups were organized in sequence of interest and by language. To determine sample size, the authors applied Malterud et al.’s (2016) ‘informational power’ model, which considers the information a sample holds along five mutually impactful dimensions: the breadth of the study aim, the specificity of the target sample population, whether the study applies an established theoretical framework, the quality of dialogue, and the analysis strategy.^54^ Considering the focused study aim, quality in-depth focus group conversations, specialized population, and applied conceptual framework, the research team concluded data collection after 16 focus groups.^54,55^ At this point, clear patterns had emerged across the discussions, and the study’s informational power was deemed sufficient.

In total, 50 adults participated in the study. Figure 1, below, summarizes the demographic characteristics of participants. Three focus groups took place in French and 13 in English. Participants came from eight Canadian provinces: Alberta (n=5), British Columbia (n=6), Manitoba (n=2), Newfoundland (n=1), Nova Scotia (n=2), Ontario (n=14), Quebec (n=18), and Saskatchewan (n=2). Most were women (n=47) and middle-aged or senior (40-70+, n=45). Racially/culturally the group was largely White (n=42) with a subset of the group identifying as East or South Asian (n=5). Within the Canadian context, the participant group was largely above average in terms of educational attainment: 90% were postsecondary graduates compared to 57.5% of Canadians.^56^ This was similarly true for income: not everyone responded to this question, but of the 40 participants who responded, 36 were in the top 50^th^ percentile, and 18 in the top 10^th^ percentile.^57^ In terms of cancer diagnosis, a majority had breast cancer (n=30), followed by lymphoma (n=4), thyroid, ovarian, and colorectal cancers (n= 3), leukemia (n=2) and uterine, prostate, lung, anal, bladder, skin, bone, and gall bladder cancers (n=1). Two participants had more than one type of cancer diagnosis. Thirteen participants had rare cancers. Notably, participants were in different circumstances in terms of their cancer status; 17 reported having cancer currently and 33 were in remission or recovery. Of those 33, nine had transitioned into remission or post-treatment survivorship within the last year and 16 in the last 2-5 years.

**Figure 1.**
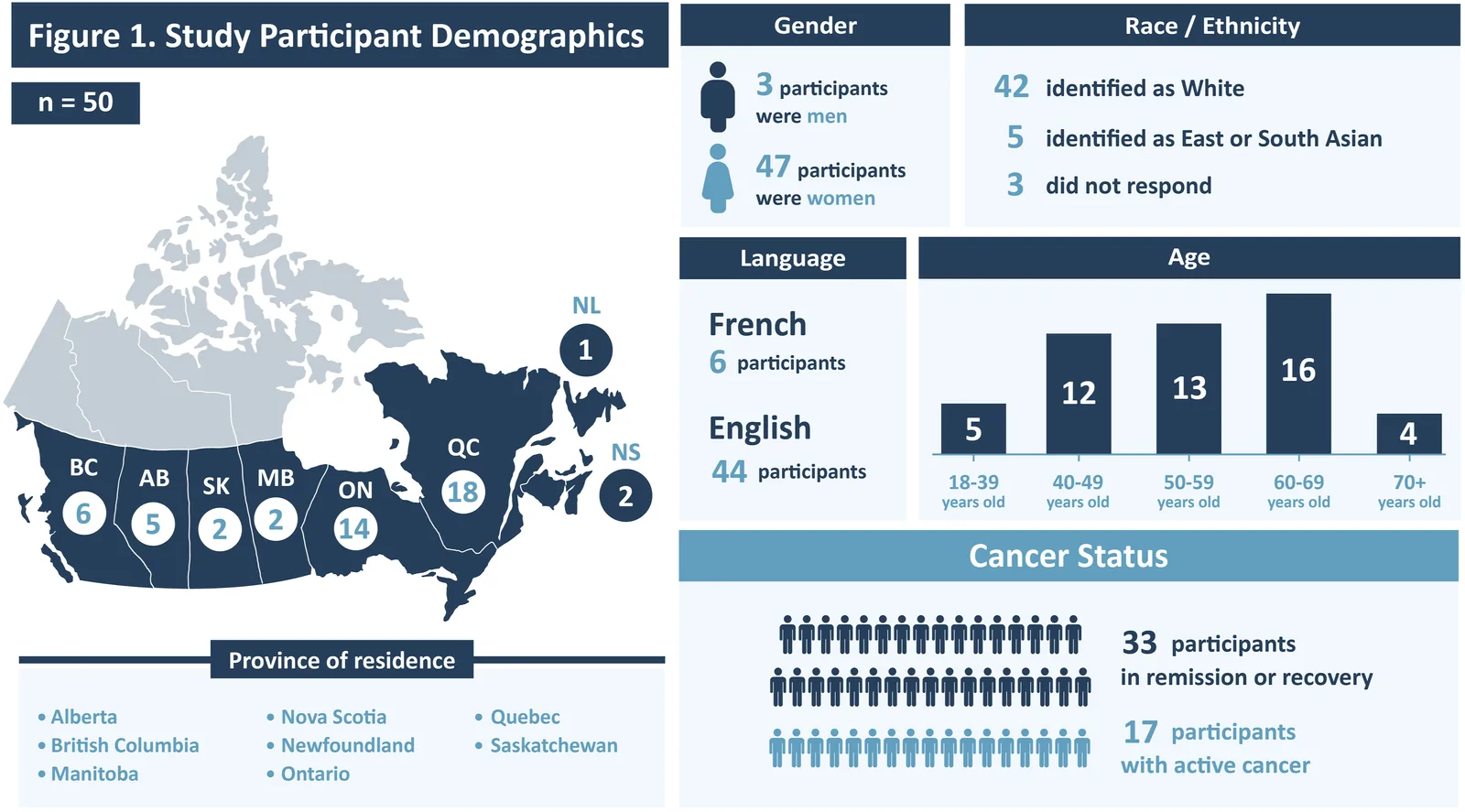
Summary of study participant demographics.

### Data collection

Focus groups were chosen as a method for this study because they bring participants into interaction with each other, providing for the exchange of perspectives, capturing a range of experiences, and generating spontaneous and refined ideas.^47,48,58^ Focus groups are conducive to sensitive topics, such as cancer, as participants are among others with shared life experiences and can support each other in what is shared.^47,48^ Hosting online was part of the study’s accessibility measures as participants could attend from their own homes. The online format has been found to be further conducive to sensitive topics^59^ and precludes any possible environmental exposures considering participants’ medical vulnerability.^60^

Between September and December 2022, two team members led a total of 16 synchronous online focus groups. Each focus group had two to five participants. Exceptionally, one of the focus groups consisted of only one participant due to the last-minute withdrawal of other participants. Focus groups lasted approximately two hours and were semi-structured. They began with introductions, a discussion of the project, a review of the consent form and establishment of verbal consent, and an ice breaker. The main topics explored were: 1) experiences with and relationship to the use of the internet for cancer-related information and support; 2) barriers and information gaps; and 3) content and features that participants would see on an ideal Canadian web portal. The first two topics are standard in the literature on the subject. The third, asking participants to describe ideal features, was done purposively as a form of “fictive scripting,” to encourage reflection on possible futures that work past existing barriers and gaps towards more responsive and patient-centered resources.^25,61,62^ Facilitators provided various questions surrounding the three different themes (see Appendix 1-Focus Group Guide) but adjusted and adapted the discussion according to the number of participants, to encourage participant interaction, to ensure balanced speaking opportunities for everyone, and to follow the conversational flow. The discussions concluded with debriefing of the study, as well as time for questions, comments, and participant feedback. Participants were remunerated for their time and contribution to knowledge. Facilitators met after every focus group and took notes on their observations and recurring themes.

The discussions were recorded with the consent of the participants. A professional transcription service produced transcripts of the audio recordings. To better safeguard confidentiality, the research team used its own recording devices rather than the recording system of the video conferencing software. Only the authors and the company who did the transcription service under a signed confidentiality agreement had access to the audio recordings. Transcriptions were then cleaned by the authors of any potentially identifying information.

### Data analysis

Interviews were analyzed via qualitative content analysis, a systematic method for identifying, examining, and summarizing repeated patterns of manifest, contextual, and latent meaning within texts.^49^ Four members of the research team analyzed the focus group transcriptions using the qualitative analysis software NVivo (1.7). Descriptive content analysis relied on an inductive, iterative approach with codes and themes derived from the data.^49,50^

First, drawing on their facilitation notes, two authors developed broad content areas that reflected the general topics discussed in the focus groups. Using these content areas as an initial organizing framework, two authors independently coded half of the transcripts, iteratively developing and refining codes and sub-codes.^63^ Codes were generated inductively and at a semantic level to stay close to participant priorities and meaning-making. The coders met regularly to compare interpretations, discuss coding decisions, and review areas of disagreement. Where coding discrepancies arose, an additional team member was consulted and discrepancies were resolved through discussion and consensus. Through this process, the research team refined the coding structure, clarified codes and sub-codes, and established a shared codebook.

The remaining transcripts were then coded using this codebook, with three team members alternating coding and validation roles to enhance analytical rigor and consistency. As a final step, two members of the research team reorganized and synthesized the coded categories into themes relevant and important to the research aims, in preparation for presentation in narrative format.^49^ Supporting quotes presented in the article were translated from French to English where relevant and cleaned of potential identifying information, moderator interjections, and dysfluencies.^64^ Consistent with the study’s commitment to centering patient agency and lived experience, a combination of short and longer excerpts was purposively included to preserve context and nuance, maximize participant voice, and allow readers to engage richly with participant accounts.^65,66^

## Results

The Results section engages with three interrelated themes in participants’ use of the internet for cancer information and support: seeking support, navigating the digital landscape, and future horizons. Together, these themes capture what people are looking for via the internet, what happens when they try to find it, and their concrete ideas for a web portal for CRIS that could sensitively and effectively meet their needs.

### Seeking support: Reasons for and factors in engagement

This first section concerns what participants were looking for and factors influencing how much they engaged with the internet for CRIS.

#### Support sought

The people in this study centrally used the internet to ease and process their experience of cancer through information, peer support, tools, and resources. Participants spoke to the profound impact of a cancer diagnosis on their lives:

*You feel like a whirlwind. Nothing works anymore. Nothing makes sense anymore. [You think] ‘why did this happen to me?’ We all experience this, just like [P31] said, this upheaval from the news. It takes small steps to come back from that. Because when you find out you’ve got cancer, inside, it’s like, well, your body is betraying you. In any case, that’s how I felt (P30)*.

For many, having information was a means to restore a sense of agency: *“For me, knowledge is power, like a lot of people say. And it reassures me”* (P36). Notably, they contextualized their control as limited within the uncertainties of cancer, but this did not take away from the value of information and knowledge for them: *“It’s very difficult, it’s very frustrating, it’s very emotional, and it’s very hard. It doesn’t matter what stage you’re at, it’s awful… To get the information means you have a small bit of power in your hands, even if it’s not very much, it’s like, okay, I know this, this is [what is happening] at the moment”* (P10).

Participants looked up a wealth and range of information about their specific cancers, including symptoms, prognosis, treatment protocols, options, and side effects, potential clinical trials, and medical research. P17: *“It just helps me as a patient to feel more informed and make more informed decisions. I feel like I’m more involved than just… being directed around the hospital. It’s more of a personal experience and I feel more empowered”*. In addition to finding medical information, participants used the internet for information on what to expect and how to navigate the medical system: *“Each province has a different way of running things. And, you know, for a lot of us who haven’t necessarily had experience of having to go through a hospital system, it can be quite overwhelming in the beginning. What’s next, where do you go, and such”* (P10). Participants further expressed that the internet was a valuable source for gaining tools and resources for their daily living in areas of symptom and pain management, diet, exercise, mental health, sleep, and so on: *“It’s also looking for those little things, those tips, that might make your life a little easier”* (P3). They searched for supplementary healthcare providers - such as counselors, physiotherapists, kinesiologists, and occupational therapists - specialized in working with people with cancer, who could help them meet their holistic health needs. Finally, participants used the internet to find peers with shared life experience to talk to and get advice from: *“There’s groups in Canada for people with cancer under forty, so I was a part of those groups as well. There was some stuff that I was experiencing that some people even over forty might not relate with, particularly impacts on fertility and stuff like that”* (P14); *“I often do the research on social media sites, so Instagram and Facebook. When there are people who have already experienced this disease or whatever I need, I follow them. I can learn a lot this way”* (P18). Connecting with other people with lived experience of cancer over the internet helped some participants feel less isolated: *“When I was diagnosed, I was really young… being able to connect with other young folks who were going through the same thing has been truly, no exaggeration, life changing for me”* (P19). Overall, participants wanted to feel they did everything they could: *“I don’t want to second guess…it’s my life, and I feel like having more information means that I’ve done everything I can do to manage my own health”* (P2).

#### Varied engagement

Participants primarily framed the internet as a tool to supplement HCP communication, and there was a broad range in frequency and interest of use. A few people did not have the internet at home, limiting their access*: “I think it’s really important, but I’ve had problems accessing it because I had to pay, and it was a bit too expensive and a bit too complicated to do so. Because of that, I’ll go to libraries if I need it”* (P18). Those participants who were close to HCPs or people with lived experience of cancer tended to prioritize those supports over online resources: *“I check in with my community first, whether that’s my family, my friends, or other folks I know who have had a similar diagnosis to me or have done similar treatments”* (P19); *“I have a group of doctor friends who I can reach out to”* (P32). Conversely, other participants did not have these types of personal supports in their lives or felt that their close ones did not necessarily have the know-how or capacity to help: *“So many friends, family, and people, they don’t know what to say. They care, and their intentions are good. But you do, you wreck their week, you burden them, and they also have literally no idea how to help you”* (P22). Notably also, participants described using the internet more during certain stages of the cancer trajectory, particularly right after diagnosis, when starting treatment, when making major medical decisions, and during recovery.

Access to, trust in, and communication with HCPs was the most significant factor in extent and nature of internet use for CRIS. Some participants preferred to keep their HCP as their sole source of information, finding this comforting. Many people had also been actively advised not to use the internet for cancer information by HCPs, and while most engaged anyway - “*I do check. I can’t help it”* (P33) - a few refrained for this reason - “*I was told by the oncology department to not look at the internet as much as possible. So, I didn’t”* (P5). Notably, those participants who had HCPs receptive to internet use for CRIS described the internet as supplementing their care: *“There were points where my oncologist would even say to me, ‘Have you asked on the Facebook group?’ Because I was only the sixth person to be treated with my type of leukemia here”* (P14).

Many participants expressed that their HCPs were not easily accessible, and that there were communication limits because of how busy their doctors were: *“They are stretched to the point of transparency. I very often did not see the same oncologist. I would have an online appointment, and it would change to two different doctors before the appointment actually happened”* (P49). Internet research thus filled a gap when there were delays and barriers in speaking with HCPs: *“That wait to see an oncologist stressed me out. So, I used that time to calm myself down by reading as much medical research as possible”* (P13). Access issues were compounded for those living in more rural and remote areas: *“Anyone who lives far from a large urban center generally is disadvantaged…Here they are overwhelmed. They have a lot of cancer cases in the region and very few resources”* (P50). Finally, for those participants in remission or recovery, many no longer had HCPs to communicate with at all: *“Once you’re cured or in remission, then they’re just like see you…So you’re on your own, and that’s when people are very prone to start Googling things I think. Because…you still have to treat the symptoms of the act of curing your cancer”* (P37).

Those participants with less trust in their healthcare team or system were also more likely to use the internet extensively for CRIS. Sometimes mistrust stemmed from past adverse experiences: *“I search for medical information because I learned that if you don’t, it’s at your peril”* (P26). Other times this cautiousness was, again, tied to structural strains on the healthcare system:

*Right now, the situation is really – I’m just going to say it’s really devastating with respect to care. I did not feel that I was being well cared for and there were things missed from my medical team…Everybody’s very overworked and things get missed. And so, I needed to be a very well-informed medical consumer in order to have me not be the thing that got missed* (P21).

Thus, the emphasis for some participants was on the need to self-educate via the internet to effectively *“be your own advocate”* (P28) within the medical system. Overall, participants had a nuanced and multi-faceted relationship to internet use for CRIS and understanding of its role in their lives, with one participant describing it as *“both a blessing and a curse”* (P47).

### Navigating the digital landscape: Gaps, barriers, and strategic approaches

The second Results section covers participant knowledge and experience navigating the internet to try and access CRIS. It concerns practical barriers and gaps people are encountering on the internet and strategies they are employing to find and screen information.

#### Encountering gaps and barriers

##### Accessibility and availability

Availability gaps and accessibility barriers impacted participants ability to access CRIS. First, some described navigating the sheer volume of information on the internet as overwhelming:

*You’re desperately trying Dr. Google. And I mean, it was like a nightmare. To not have any sort of, here’s* www.mycancer.com*. To go on [the internet] and find all the information that you would need…having to sieve through all that information, it’s not fun* (P10).

Second, the sensitivity of how information was framed and its emotional impacts were also an element of accessibility. Some participants wanted to look up information but found the experience too upsetting: *“It’s too demoralizing”* (P50). Third, participants expressed that certain types of information were more difficult to find, particularly for rare cancers, having cancer as a younger adult, whole person support, and recovery from cancer: *“My own disease is pretty rare. So I couldn’t find much. There is very limited information available”* (P8); *“There’s a real dearth of information regarding survivorship”* (P1); *“One thing that is really lacking is looking at it from a holistic care sort of lens…looking at the individual, the person beyond the body that is facing this disease”* (P46).

Many participants were interested in finding the latest research available on their specific cancer but encountered difficulties with specialized medical writing and terminology. Even considering that our participant group was highly educated, medical research is a specific academic discipline: *“Not everybody is science literate”* (P25). Paywalls for academic journals were also identified as a barrier: *“Sometimes you don’t have access to the study. You get a block or you get a summary unless you subscribe”* (P2). Accessibility issues were compounded for individuals experiencing “brain fog” due to chemotherapy, which affected their cognitive capacity to follow and engage with complex information: *“In the throes of chemo you can’t process the scientific journal information. Some of it was just too hard to understand for me while I was going through treatment”* (P22). At the same time, multiple participants pointed out that online resources catered towards a public (rather than research) audience often contain over-simplified information and wished for a middle-ground of accessible yet substantive information:

P1: *The Canadian Cancer Society [website], they have all this beautiful information, but it’s so high level that it doesn’t really do anything. [In contrast] the NHS has a really nice website that goes over all of the different sorts of cancers…It gives you the epidemiology, the really intense stuff that you see in publications, in a beautiful format. It’s also written in plain English….but it’s just not relevant to the Canadian context.*

P2: *The other thing for me is that I want the research summary. I want the conclusions from those studies, and I want to know a bit of background about them because that’s where I make my judgement…I don’t like simplified little diagrams and all that, that drives me crazy.*

Thus, despite interest and active efforts, participants could not always access the cancer information and research they sought online.

##### Ads and misinformation

Participants also spoke about being exposed to a considerable volume of ads and misinformation on the internet: *“You look up something and you get bombarded by all kinds of ads for all kinds of treatment…Everybody’s got something to sell on the Internet. Somebody’s trying to make money and profit from all this. I don’t want to be a part of that”* (P4). Most were aware and alert to misinformation -*“Somebody sent me information about how I had to drink liquid oxygen”* (P13)- especially on social media - *“I take it with a grain of salt”* (P3). Sometimes these overtures could feel insensitive: *“The amount of people when I was first diagnosed that would send me articles like ‘eat more asparagus’ - I was like, I don’t think they’re getting the situation I’m in right now”* (P14). At the same time, some people expressed how the experience of cancer and the vulnerability and fear it brings led them to consider services and products even when they knew they had predatory pricing or were fraudulent:

*You’re so damned scared that you almost want to do some of that stuff. I wrote naturopaths and I got $40,000 quotes from two of them. For $39,000 and $45,000 they can cure my cancer. I was scared, I wanted to be around for my kids. I was very tempted to fundraise for the money”* (P22).

Overall, ads and misinformation were described as a preponderant, frustrating, and potentially harmful element of seeking CRIS online.

##### Privacy

Participants expressed mixed feelings regarding their privacy online. On one hand, many valued sharing personal health information in order to help out other people with cancer. On the other hand, they also expressed various levels of concern about their privacy and the potential implications of their personal information being shared and tracked online. Each individual had different thresholds for the amount and type of information they are willing to share. For many, financial data, such as banking information, was more critical than medical data. Consequently, some advocated for easier access to medical records, given the relatively straightforward access of other sensitive information:

“*If I can get an app with all my banking information, and all my credit history where you can see where I’ve worked and lived and all the money I’ve made…why can’t we have an app for our medical history?* (P38)”

Others were reluctant to allow access to their medical data. For instance, some participants hesitated to disclose their medical conditions with others because they feared it may lead to being treated differently: *“I don’t want the pity, I don’t want the look”* (P4). Several were concerned about data misuse -*“It’s scary to share, because anybody can use that against you”* (P33) - or use by private companies - *“I do have a fear that some of this information can be used by corporations”* (P2). Some participants also reflected that there may be a generational divide, with the younger generations being more willing to share their data compared to older generations: *“I’m old fashioned, in the sense where I don’t understand how some people share very personal data”* (P17). Finally, multiple participants expressed confidence that their data is well protected due to the existing safeguards:

*“I know that there are quite a few laws in place now to protect folks who are carriers of certain genetic mutations in the event of any sort of discrimination that may occur. So, I feel comfortable with that, because of the legal protections”* (P19).

Participants also discussed the strategies they have employed to safeguard their privacy online. This included limiting the amount of personal information they shared on the internet - *“I don’t disclose an awful lot about myself on the internet or on social media”* (P47) - downloading antivirus software - *“On my laptop, I’ve got McAfee that runs in the background”* (P34) - and changing privacy settings - *“Figuring out the privacy settings on each of my social media platforms empowered me”* (P3). However, despite these safeguards, they were often faced with having to choose to access important information over maintaining privacy: “*I do try to protect my information but at the same time I’m aware, just by accessing websites that some of my information is being tracked”* (P48). Finally, many acknowledged that while most individuals are trying to maintain privacy, some degree of information will inevitably be tracked regardless of their efforts: “*I think that’s just the direction we are moving into…I gave up the notion of any kind of privacy a long time ago”* (P20).

#### Strategically sourcing and critically evaluating information

Participants described various approaches that they have employed to navigate the gaps and barriers they encounter on the internet. Some also brought up feeling underestimated in their skills in this respect: *“I feel our medical system automatically jumps to us being too stupid to look up proper sources”* (P38). Participants tended to place their trust in well-established and reputable sources, including various cancer society and medical organization websites: *“WebMD is a pretty good one. The National Cancer Research Institute is another one I go to. The Cancer Research Society, sites like that”* (P20). They shared strategies they employ to critically evaluate the information they come across on the internet. For example:

P19: *I will just start by evaluating the source. Who’s publishing this information? Who’s writing this information? What are their credentials? What are they citing in their research as proof? How relevant is that proof? How recent is it? Is it from 2019? Or is it from 1998?*

P21: *Yeah, I would agree and I would add – is it peer reviewed? And if so, in what journals?*

The perceived professionalism of a website, including spelling or grammatical errors, website design, domain extension (i.e. using ‘.com’ versus ‘.org’), and linking other reputable sources were also important in critically evaluating the source:

*I look and see if the site is well maintained, at the design integrity. Does it look too flashy? Does it look too basic? Are the links easy to access? Are there links to other well-known websites?… Just overall professionalism, because there are some sites out there that right away, right off the bat, you look at them and you’re like that’s sketchy* (P20).

Several participants noted that they would check multiple sources to validate information: *“If I come across something a bit weird, I’ll do some more research to see if there are other sites that say the same thing, to see if there’s any information that supports the first thing I saw”* (P18). Finally, while critically evaluating the information they chose to consume on the internet, participants also demonstrated practices of agency by only accessing resources based on what they were emotionally prepared to read: *“I knew I wasn’t ready to see a certain amount of information before my surgery. I had to go in little steps and let my brain get okay with everything”* (P24).

### Future horizons: Ideas and ideals for therapeutic web portals for CRIS

The third and final section covers participant ideas for an ideal pan-Canadian therapeutic web portal for cancer information and support. It answers the question: given all the above, what could be better?

#### Holistic health information: Physical, practical, and psychosocial needs

Participants expressed similar needs when it comes to the content that they would like to find in a therapeutic web portal for CRIS. They envisioned an ideal portal fulfilling two essential overlapping functions: meeting their holistic health needs, and filling gaps in types of cancer information and supports that are currently hard to find or poorly resourced on the internet.

First, participants emphasized the importance of comprehensive high-quality information covering all stages of cancer, from diagnosis to recovery (i.e., adjusting to life after the disease) or end of life: *“The stage that the patient is at, and what they need at each stage”* (P24); *“Not just the medical treatment of the disease, the whole person”* (P35). Within this broad scope they highlighted the need for both factual information (e.g. details on diagnosis, types of treatment available, side effects) and processual information (e.g. how to navigate and self-advocate within the hospital system, what to expect in treatment, how to communicate with friends and family). They also emphasized the importance of providing both general information and more detailed resources for those seeking deeper engagement.

Information on both recovery and end of life care was noted as very under-resourced by participants throughout the focus groups. Many participants described the post-treatment phase as especially challenging due to gaps in available information: *“I would say, I am finding it harder now, after the cancer, than when I was in it. There’s a lot of information missing to make me comfortable living with the collateral effects”* (P18). Participants expressed that life after cancer information could help with reintegration, navigating side effects and bodily changes, looking for work, disclosure, minimizing risk of recurrence, future planning, and various other aspects of integrating their experience of having had cancer into their lives. At the same time, they also asked for compassionate information, resources, and whole person supports for those with terminal cancer.

Participants also stressed the significance of access to current and emerging research and innovation: *“The latest research, latest treatments, I would welcome that”* (P33). They emphasized that the research information be *“geared towards various backgrounds, different levels of expertise”* (P4). They also expressed interest in information about available clinical trials: *“Clinical trials can be difficult to know about, especially if your cancer is rare. And so that would be something that a specific cancer site could do; provide a link to the clinical trials for that specific condition*” (P36).

Beyond medical and research information, participants wanted information on complementary and supportive resources addressing psychosocial and practical dimensions of care. These included services such as physiotherapy, massage therapy, and psychological support - *“I don’t believe there’s anybody who has a serious bout of cancer that does not need therapy”* (P37) - as well as guidance on nutrition, exercise, sleep, physical comfort, and navigating the financial burden of cancer:

*Some people go into large amounts of debt - when they’re dealing with a diagnosis and trying to get treatment. Maybe not from the drugs themselves, but from not being able to pay their bills or whatever else from not being able to work full time, from being sick half the time. I think…learning how to prepare for that would be helpful* (P38).

Support groups were considered essential for emotional expression and shared understanding: *“[You] can voice out all your fears. Not just physical…but also your emotional fears. Because it is very stressful, knowing that you have cancer. And you really need people to understand you”* (P32). Equity considerations were also central. Participants stressed that an ideal portal should be inclusive of diverse populations and underserved communities:

*It has to bridge very important gaps from a health equity lens. So, if we’re talking about folks who have challenges in accessing care and support services due to being in remote or rural communities or being in communities that are otherwise traditionally underserved or underrepresented… I think that it would be very important to be inclusive of those things and be mindful of speaking to all audiences because, again, cancer just does not discriminate.* (P46)

Finally, participants also asked for the portal to have interactive features that would enable more personalized support, such as the ability to send questions to HCPs or patient navigators. These features were seen as critical for fostering reassurance, guidance, and connection: *“Having someone you can actually talk to and who will talk back to you. Make you feel that it’s okay, ‘we have resources, we can put you in touch with this or that’, [tell you] ‘this is what you should do’.”* (P12).

#### Portal design considerations: User-friendliness and accessibility

In addition to providing the right content, participants emphasized that the presentation of information plays a crucial role in accessibility and user-friendliness. There were two key takeaways regarding portal design from focus groups. First, that cancer patient experiences and needs are not monolithic and that ideally a portal could be applicable and useful to as broad a range of people as possible, particularly those who are currently underserved: *“Given that you have a multitude of needs, you need multiple different ways of conveying the information”* (P50). Second, that the experience of cancer should be considered within portal design. This includes its stated value in vetting, streamlining, and curating information - *“What’s available searching on Google, some of it is really reliable, and some of it is quite frighteningly unreliable. And there needs to be some way to sift that”* (P8) - and the emotional, physical/energetic, and cognitive accessibility needs that accompany cancer - *“Friendly information, if you know what I mean. Not so technical and scientific. Friendly, accessible, comforting information. I think that would help a lot of people, whatever your cancer type is.”* (P42).

A frequently mentioned suggestion was to use concise plain language summaries to ensure that the shared information was easily understood by most individuals. Some participants suggested having separate sections for layperson summaries and scholarly articles to cater to different needs:

*There are times you know that I feel that I’m on point, that I can focus, that I can follow through with a scholarly article. And there are other times that things just don’t enter…if people don’t want to feel like they’re being patronized or made to feel like they’re stupid then there’s the scholarly article route and then for people who just want to know and just want to digest information easily, there’s the other route. So, I think having both is really essential* (P20).

Numerous participants recommended organizing the information into sections in order to prevent overwhelming users and allow them to curate the information according to their preferences:

*Like a funnel. [Having] the landing page of what are you here to find first. And then it could guide you through as you go deeper. Say you are looking for statistical information or research information, it can take you deeper and deeper as you want to find more* (P6).

Enabling more personalized navigation of portals was also viewed as a means to accommodate distinct ways of coping and processing information:

*Everyone goes through this process differently. Some people want to be in denial, some people want the information, some people want it framed nicely, those sorts of things. If you want to meet everyone’s needs and where they’re at, I like that idea of having those different stages of information…Maybe starting with something simple, but then if they want more information, being able to link them to more statistical information, more peer-reviewed journal articles or something like that”* (P35).

Therefore, various formats were recommended, including links to other resources, videos, graphs, statistics, decision trees, and more. Participants also highlighted the importance of carefully considering the way information is framed and displayed during the design, since it can have a psychological impact: *“I really want a site where I won’t feel depressed about my cancer…it has to be a positive site”* (P32). Table 1 summarizes participant perspectives on the ideal components and design of therapeutic portals for people living with or beyond cancer.

**Table 1.** Participant perspectives on the ideal components and design of therapeutic portals for people living with or beyond cancer.

Components	Information Systemization and Curation	• Information on various cancer types, including rare diseases, and considering different demographics and life circumstances
		• Information for each stage: what to expect, treatments, daily living, links to further supports
		Diagnosis
		Treatment
		Remission/recovery
		End of life
		• Decision trees
		• Information for family and friends
		• Links to credible and reputable websites for more information
	Research Information	• Plain language summaries of peer reviewed studies
		• Studies in multiple languages
		• Information on ongoing and upcoming research
		• Information on clinical trials
		• Genetic testing information and genetic research
		• Possibility to give feedback/have a suggestion box
	Resource Accessibility	• Links to helpful services and advice: patient portals, supplementary health services, cancer-informed exercise and leisure activities, financial assistance, fatigue and pain management etc.
		• Information on local HCPs and specialized HCPs including reviews and availability
		• Access to HCPs to follow-up with during cancer and in remission/recovery
		• Support groups
		• Section for questions: FAQ, virtual assistants
Design	• Create account (username and password for security purposes)	
	• Onboarding process to help navigate portal	
	• Prioritize user-friendliness in design	
	• Funnel & compartmentalize information – according to cancer types, stages of treatment, type of information, extent of detail etc.	
	• Use concise accessible empathetic language – sensitive to the experience of using the portal as a person with lived experience (PWLE)	
	• Mix of information types (layperson & scholarly, textual & visual, quantitative and qualitative etc.)	
	• Optimize for multiple devices/browsers	

## Discussion

The Discussion section develops three interconnected areas arising from study findings: patient agency and internet use for CRIS, equity and access factors, and the potential of structured online resources. Together these areas situate participants’ experiences within broader debates about cancer care and digital health, highlighting both the constraints of current resources and the opportunities for more responsive, inclusive, and patient-centered tools.

### Patient agency and internet use for CRIS

Study findings highlight that people living with or beyond cancer come to the internet with a multiplicity of experiences and needs.^4,31,41,67^ Within this study, participants had different cancer situations, life circumstances, social locations, and levels of scientific and internet literacy. This study moves forward from earlier literature that questions whether patients should use the internet for CRIS. Following our conceptual framework, an agency-based understanding of the internet as a resource starts with the question of how to best optimize online resources for people living with or beyond cancer. To help shape effective internet tools and venues that facilitate patient agency, it is important to listen to patient needs, concerns, and considerations.

Reasons for and factors in internet use for our participants echoed previous literature.^10,13,31,42,68,69^ For many participants, having access to CRIS helped them gain medical knowledge, a sense of control and autonomy, and sometimes reassurance. At its best, the internet helped people ease and process their experience of cancer,^42^ connect to peers with shared experience,^70,71^ and better navigate the healthcare system. Those who used it often really valued it as a resource. They not only acquired information but acted upon what they learned within their lives. Returning to our conceptual framework, internet use for these participants can be seen as a practice of agency within the ‘discontinuities and disruptions’ of cancer. At the same time, it is also important to respect that not everyone will want to use the internet for CRIS, and that choosing not to can be an act of agency in of itself. A few participants fell in this category. There is increasing pressure on patients to be very actively engaged and assertive within their healthcare but, for a variety of reasons, this is not what everyone needs or wants.^70,72,73^ In the experience of cancer, agency can mean both taking and letting go of control.

This study contributes a holistic understanding of the cancer experience and CRIS. This holistic understanding is valuable towards the content and design of emergent online tools such as therapeutic portals. This includes understanding the need to exercise agency and dignity in multiple dimensions of life (e.g. physical, psychological, social).^53^ It also includes considering the emotional experience of cancer when meeting informational needs; the two cannot be neatly separated^33^ and are not separated by people with lived experience of cancer. This has been, but does not need to be, a disconnect between patient frameworks and those of other stakeholders shaping internet resources for CRIS.^74^ Initiatives and tools for patients can both be informational and sensitive and relevant to experience of cancer.

### Access and equity factors

Existing literature acknowledges the potentially empowering effect of the internet, but also that this effect cannot be seen in isolation from other sources of information accessible to patients, their relationship with their healthcare providers, and the wider context of healthcare and information.^4^ Three access and equity factors that impacted internet use for CRIS among our participants were user demographics, missing information, and communication with healthcare providers.

#### User demographics

The demographics of participants in this study were consistent with what other studies have found: that internet users for CRIS are more likely to be women,^12,42^ have higher education,^12,35,72^ and/or have breast, gynecological, or prostate cancers.^70,75–77^ Similarly, internet users for CRIS and participants in online focus groups tend to have a baseline comfort level using digital technologies and online resources.^34,35,78,79^ It is significant that the internet has been a helpful tool for CRIS for the demographics included in the study, and the next step would be to take initiatives in resourcing it to be helpful to people from as many backgrounds and life circumstances as possible. For those from historically marginalized and underrepresented groups, this will necessitate proactive efforts. Several systemic inequities were raised by participants in this study, including that not everyone has internet access, that within Canada there is less healthcare resources and access to information in rural/remote locations,^38,80^ and the importance of sensitivity and relevance of online resources to members of equity-seeking groups.

Within the broader literature on the topic, this research contributes perspectives from older users^16^ and from people with rare cancers,^72^ both of which are underrepresented. Older adults in this study were actively using the internet; there were not substantive differences in how they discussed online resources or in their internet literacy. This could be in part because the sample was largely highly educated and/or may indicate older patients being underestimated in their interest and capacities as has been seen in other studies.^16,81^ However, there were some differences in the older adult participant group subset in that some were more hesitant to ask doctors questions and/or more cautious about privacy online relative to the group as a whole. Some of the advantages of the internet as a forum for CRIS, such the value of being able to access it from home, were also compounded for older participants, who have additional accessibility barriers. In terms of participants with rare diseases, they described exacerbated barriers to finding information, given the low incidence of their conditions (see section below), and described online peer support groups as essential to this end. Overall, our findings emphasize that designing equitable CRIS resources requires moving beyond assumptions of a generic patient user toward proactive inclusion of underrepresented populations.

#### Missing information

The fragmented and dispersed information currently available to cancer patients further exacerbates inequities in access to specific types of information, an issue highlighted in patient focus groups and in existing literature. Certain resources remain particularly scarce for patients such as information on rare diseases,^67,72^ recovery,^82,83^ end-of-life care,^84^ the distinct challenges faced by younger adults under 40,^82,85^ and holistic support including mental health.^86^ Within our participant group, the need for better resourcing of rare disease and recovery information was particularly salient.

People with rare cancers often struggle to find reliable, disease-specific information, leading to uncertainty in decision-making and long-term health planning. It is necessary to incentivize healthcare professionals and researchers to prioritize rare diseases and personalized care, while also increasing efforts towards developing rare disease education initiatives, which remain insufficient.^87^ Although rare diseases have gained recognition as a global public health priority, existing resources still fall short in adequately supporting patients with these conditions and addressing their unique informational needs.

Advances in genetic screening, molecular profiling, and increasingly sophisticated genomic and transcriptomic approaches have expanded opportunities for personalized cancer care.^88^ However, these developments also intensify existing informational gaps, particularly for patients with rare cancers who often face limited evidence bases and uncertain treatment pathways. Many genomic findings continue to carry uncertain or evolving clinical significance and lack standardized management guidelines^89,90^ creating additional challenges when communicating results. To address this, Peterson et al. (2018) advocate for a multilevel approach encompassing provider training, evidence-based decision support, and the use of portals and other tools to enhance communication and counselling.^90^ This reinforces the need for digital platforms capable of delivering personalized, patient-centered information and support.

Finally, individuals in cancer recovery often face ongoing health issues and significant informational gaps. As patients transition from active treatment to survivorship, their informational needs evolve towards ongoing monitoring, rehabilitation, and long-term health maintenance.^11,91^ Better addressing these needs through comprehensive, personalized resources could significantly enhance the recovery experience, providing patients with the knowledge and support necessary to navigate the complexities of life after cancer.

#### Communication with healthcare providers

Findings from this study align with previous research wherein internet use for CRIS supplemented communication with healthcare providers^7,8^ and participants found it useful to this end. Further, among participants whose doctors were open to discussing what they found online and answering related questions, internet use enhanced their healthcare experience. For example, in understanding and combatting their diseases and in accessing treatments and complementary supports. Indeed, healthcare providers can help guide patients toward credible high-quality resources and integrate online resources into care pathways.^13^ However, many participants had HCPs who were hesitant about patient use of online resources for CRIS. This hesitancy has been noted in other studies.^13,25,92^ The repeated finding of patient use of the internet for CRIS and HCP reticence to discuss it, indicates a need for HCP adaptation to the phenomenon.

There was a notable relationship between participant internet use and service gaps, including barriers in access to HCPs^4,42^ and long waiting periods for test results and treatments.^93^ There was also a relationship between medical mistrust and internet use. On the one hand, internet use was experienced as a valuable empowering tool that helped offset these problems, and this trend is a testament to its growing usefulness as a resource. On the other hand, the internet is not a solution to structural resource strains and those with the least contact with HCPs and/or trust in the medical system may be the most vulnerable to predatory ads and misinformation.^43,94,95^ The structural strains subtheme that emerged from this study points to the need for policy and resourcing changes in the Canadian healthcare system to help increase patient access to physicians.

### Structured online resources for patients

The gaps and barriers participants encountered navigating the open internet for CRIS underscore the need for accessible, relevant, and effective tools that can help meet the needs of people living with and beyond cancer. This study contributes to the literature on patient perspectives on the value, design, and utility of structured online resources. Such resources can facilitate and reinforce patient agency, even in circumstances where control over health outcomes is limited.

#### Volume of information online

One of the biggest challenges in accessing cancer-related information is information overload. The sheer amount of information available online makes it challenging for patients to filter and identify relevant and credible information.^14,96^ In fact, according to a scoping review, cancer studies usually focus the most on health information overload,^97^ highlighting the pertinence of the issue. The core problem is not so much the high volume of information but rather the way it is structured and presented.^98^ As we increasingly turn to digital tools, available information remains unorganized and cluttered with irrelevant content, making it less accessible and more difficult for users to find accurate helpful information.^14,98^ While health and digital literacy can help patients navigate online content, patients should not carry the burden of navigating complex, unfiltered, and unorganized information.^72,97^ Online resources that filter and titrate information for patients are one practical solution to the general problem of information overload.^24,25^ More systemically, greater resourcing towards digital literacy and prioritization of user-friendly designs will be important.^72,99^

Participants’ desire for increasingly specific and personalized cancer information suggests that future digital platforms may also need to manage substantially larger and more complex datasets. For example, the integration of electronic health records and genomic sequencing data presents significant challenges. Managing these heterogeneous datasets requires robust data architectures and computational approaches capable of standardizing, harmonizing, and safely interpreting large-scale health information while maintaining reproducibility and generating meaningful patient-facing resources. As future digital platforms increasingly rely on population-scale genomic and clinical data, these requirements highlight the substantial infrastructural, governance, and resource investments needed to support next-generation digital health systems.^100,101^

#### Advertising and misinformation

There are currently no clear standard requirements for ensuring quality online content, which contributes to the spread of inaccurate or misleading information.^14^ The literature highlights participants’ concerns about how low-quality information can present as a barrier.^6,67^ Further, misinformation and predatory advertising must be addressed, as they can be exploitative of vulnerable patients and cause harm. In fact, misinformation is the leading cause of preventable death in America, according to the FDA.^102^ Participants, like many other individuals, were proactive in adopting strategies to assess and detect trustworthy information, but the larger issue is that they should be encountering less inaccurate content.^42^ Structured online resources can help address this issue by filtering out ads and misinformation, in favor of relevant information tailored to patient needs.

While our study focused on patients’ use of the internet and their envisioned features of an ideal therapeutic portal, perspectives on artificial intelligence (AI) integration were not explored. This represents an important area for future research given the growing integration of AI and algorithmic tools in how patients seek and engage with health information.^103^ A recent meta-analysis found that while cancer-related information across these platforms demonstrated relatively high understandability, it fell short in terms of actionability and completeness, with content found to be misleading or commercially biased.^104^ This highlights that regardless of whether patients turn to conventional internet resources or AI for health information, ensuring the accuracy and quality of the information provided will be critical. There remains a need for curated digital resources, clear guidelines and reliable tools to guide patients.

#### Sensitivity of information

As internet-based tools for CRIS continue to develop, it is essential to consider not only what content is presented, but how it is conveyed. As participants shared, the impacts of experiencing cancer and cancer treatment mediate how people engage with information about cancer. Literature has noted that some forms of content - such as statistical survival rates, descriptions of disease severity, or complex clinical language - can be distressing, particularly when not presented with care or context.^6,57,105^ In this study, participants reflected a range of needs and emotional states, underscoring the importance of sensitivity in both framing and delivery of online cancer information.

Presenting health information with empathy and awareness of its potential emotional impact is not only a matter of good communication but of accessibility. Holistic approaches that include psychosocial and practical considerations alongside medical knowledge are resonant and relevant to patient lived experience. A recurring theme in both the focus groups and the literature is the need for staging information across different phases of the cancer trajectory - pre-treatment, active treatment, remission, and beyond.^58,96,106^ Online tools designed with these experiential shifts in mind can better support patients over time, recognizing that what feels helpful or manageable at one moment may be too much or too little at another. Ultimately, creating sensitive cancer-related internet resources requires a patient-centered approach and a commitment to accessible, thoughtful design.

#### Privacy of information

The level of awareness regarding medical data protection varies among individuals, as factors like information sensitivity can influence how individuals safeguard their data.^107^ In our study, some participants felt that a breach of their banking account information would be more harmful than a breach of their medical data, and some wanted access to their medical data online. Many factors can come into play in how individuals share their data and perceive associated risks.^108^ However, most participants mentioned adopting privacy strategies and being attentive to privacy risks. Many also acknowledged having limited resources to ensure data privacy when websites use tracking cookies. Studies have shown that while the European Union General Data Protection Regulation (GDPR) has helped strengthen data protection, it remains insufficient.^109^ Therefore, increasing knowledge on risks and developing responsible data sharing approaches online is important.^110^ The range in participant feedback evinces the need for privacy and security measures within online resources for patients, especially if sensitive health information will be involved.

#### The potential of therapeutic portals

Part of this study involved asking participants to imagine an ideal Pan-Canadian portal for CRIS. While not all the ideas they raised are immediately feasible, such exercises are important for envisioning new possibilities and directions for online resources. Participant reflections underscore that therapeutic portals can play a proactive role in the cancer information landscape. An ideal therapeutic portal was described as a curated and centralized space that integrates diverse information and resources: plain language medical information, emerging research, clinical trial opportunities, and holistic supports across the cancer trajectory. This was not simply a call for more information, but for information that is trustworthy, accessible, and aligned with the shifting needs of people living with or beyond cancer.

Participants emphasized the value of portals that can bridge medical knowledge with psychosocial and practical resources. They described how a therapeutic portal could consolidate information on under-resourced topics such as rare cancers or life after cancer, while also ensuring that content is presented in sensitive, digestible ways. Therapeutic portals were also associated with relational and interactive functions. Participants consistently highlighted the value of features that would allow them to ask questions of health professionals or connect with peer communities. These kinds of interactive elements move therapeutic portals beyond the static function of simply storing or transmitting information, toward becoming dynamic resources that facilitate communication, validation, and continuity of support.

Taken together, participant visions point to therapeutic portals not as a replacement for existing online information, but as a means of restructuring how cancer information is gathered, curated, and delivered. By embedding research, clinical, and lived-experience knowledge in a single, navigable space, therapeutic portals could address the persistent problems of overload, misinformation, and fragmentation while also supporting patient agency across the cancer experience.

### Study limitations

A qualitative approach was taken in this study to prioritize rich, in-depth findings that convey the realities and perspectives of participants with lived experience of cancer.^46^ Findings are not broadly generalizable and should be understood within the Canadian context, the demographic profile of the participants, and the rapidly evolving digital landscape. Recruitment relied on self-selection, which may have biased the sample toward individuals who were more engaged, informed, or comfortable discussing internet use and online resources for CRIS.^78,79^ As a result, the challenges faced by individuals with lower digital literacy, less technological confidence, or more limited internet access are likely underrepresented in this study. The sample was also largely composed of White, highly educated women, and while this reflects common patterns in cancer research participation,^72^ it does not capture the full spectrum of patient experiences or perspectives, particularly among those facing intersecting barriers to healthcare access or digital inclusion. Future research should prioritize the inclusion of more diverse perspectives, including people from varied racial, ethnic, geographic and socioeconomic backgrounds, to reduce potential bias and better account for context-specific needs. Such efforts may support the development of more accessible and inclusive therapeutic portals. Additionally, while the focus group format enabled rich and reflective discussion, it is possible that some participants may have moderated their responses in group settings or that less confident speakers were underrepresented. These limitations highlight the importance of complementary research focused on underrepresented groups and individual experiences.

## Conclusions

The present study sought to better understand the views of people living with or beyond cancer on the role of online resources for cancer-related information and support (CRIS). In doing so, it centered patient agency and lived experience. Study participants used the internet to better understand, process, and manage their experience of cancer. For many, it played a crucial role in restoring a sense of agency, reducing uncertainty, and connecting with others with shared lived experience. They engaged strategically and resourcefully, and at best, the internet helped them attend to their care holistically and exercise dignity and choice. At the same time, participants encountered significant barriers, including information overload, accessibility gaps, predatory advertising, misinformation, and privacy concerns. Their experiences evince a need to improve the quality, diversity, relevance, and accessibility of online resources. More broadly, there is a need for greater sensitivity and responsiveness to patient cancer information and support needs.

Yet participants did not merely highlight deficiencies; they articulated a vision for better digital tools - tools that offer credible, curated, and compassionate resources, that reflect a holistic understanding of cancer, and that are designed with flexibility, inclusivity, and user-friendliness in mind. Therapeutic web portals can be one such resource, consolidating and organizing high-quality research, findings, and data to increase the reach and accessibility of cancer information and support. For developers and healthcare policymakers, findings point to several priorities for the design and governance of future therapeutic portals, including plain-language and health literacy-informed communication standards, structured curation of information, peer-support spaces, robust privacy and data governance frameworks, and the integration of medical, psychosocial, and practical resources. Patient perspectives are invaluable to the efficacy, relevance, and user-friendliness of these platforms and should continue to inform their development, implementation and evaluation. Online resources that are co-designed with patients and embedded within equity-informed frameworks can offer more than information - they can support autonomy, facilitate care navigation, and help people manage the complex physical, psychological, and practical dimensions of living with and beyond cancer. Ensuring that the voices of people with lived experience remain central is essential to transforming web-based CRIS from an overwhelming, unreliable, and exclusionary terrain into a supportive, empowering, and human-centered ecosystem.

## Supplemental material


Supplemental Material - Nurturing connections: Patient insights on online resources for cancer information and support
Supplemental Material for Nurturing connections: Patient insights on online resources for cancer information and support by Terese Knoppers, Sarah Bouhouita-Guermech, Ana Eliza Bonilha, Angelica Voutsinas, Marie Cosquer and Ma’n H. Zawati in Digital Health.

## Data Availability

Data is available upon reasonable request.*
