# Supplementary material for: Nurturing connections: Patient insights on online resources for cancer information and support
Source: Digit Health. 2026 Sep 17;12:20552076261475254. doi: 10.1177/20552076261475254 (PMC13724918; doi:10.1177/20552076261475254)
Supplement: Supplementary file 1 [file sj-pdf-1-dhj-10.1177_20552076261475254.pdf]

Appendix 1: **Focus Group Questions**

Semi-structured FG format, questions adapted to each group

Topic 1: Current use and perspectives on the web concerning medical information

- *Do you use the internet in your daily life?*
- *If you have concerns or questions about your health, where do you first go for information? Is it a doctor and/or nurse, the internet, a family member, a friend, etc.?*
- *For what reasons would you use the internet to search for cancer information?*
- *How would you describe the types of cancer information you search for online?*
- *If you read something online that was new to you, would you follow up with a doctor or healthcare professional? Why or why not?*
- *What amount of information do you like to have? Do you like having all the information at your fingertips or do you feel like too much information is not necessarily good?*

Topic 2: Barriers and information gaps

- *When looking for cancer information online, have you ever encountered any difficulties? If yes, what were they? Are there types of information that are easier to find than others? Do you find that easy or hard to find information online?*
- *How do you judge the quality of the information you read online? How do you assess if it is reliable or not?*
- *How do you feel about sharing personal information, especially related to your health, online? How do you decide what to share or not?*
- *Do you pay attention to or manage your privacy settings when you navigate on the internet or when you look for medical information?*
- *How do you know if information is reliable or not?*

Topic 3: Needs, desires, and expectations for an ideal therapeutic web portal

- *Have you used a web portal for accessing health information before?*
- *How do you prefer to learn new information? (e.g. reading text, using interactive features, diagrams, videos, audio, asking someone, etc.)*
- *Which aspects of a web page design are most helpful for you when you are accessing information online? (e.g. clear and visible fonts, accessible language, easy to navigate, interesting design…)*
- *What device(s) do you use to access information online? Is it convenient for you?*
- *What would you want to see on an ideal web portal? What would it look like to you?*

What benefits would you hope to get from using a web portal for cancer? How could it be helpful?
